# Salicylic acid alleviated the effect of drought stress on photosynthetic characteristics and leaf protein pattern in winter wheat

**DOI:** 10.1016/j.heliyon.2021.e05908

**Published:** 2021-01-07

**Authors:** Masoumeh Khalvandi, Adel Siosemardeh, Ebrahim Roohi, Sara Keramati

**Affiliations:** a Department of Agronomy, Faculty of Agriculture, Shahrood University of Technology, Iran; b Department of Agronomy and Plant Breeding, Faculty of Agriculture, University of Kurdistan, Iran; c Kurdistan Agricultural and Natural Resources Research and Education Center, AREEO, Iran; d Department of Agronomy, Genetic and Agricultural Biotechnology Institute of Tabarestan, Sari Agricultural Sciences and Natural Resources University, Sari, Iran

**Keywords:** Photosynthesis, SDS-PAGE, Salicylic acid, Drought stress

## Abstract

Salicylic acid (SA) is a promising compound to increase plant tolerance to drought stress, and it can affect many aspects of physiological and biochemical processes. This study was focused on the changes in proteins, photosynthesis, and antioxidant system of Sardari wheat ecotypes leave in response to the application of SA under drought stress conditions. Treatments included Sardari wheat ecotypes (Baharband, Kalati, Fetrezamin, Gavdareh, Telvar, and Tazehabad), salicylic acid at 0.5 mM (controls were untreated), and drought stress (30% of the field capacity). The results showed that membrane electrolyte leakage, and lipid peroxidation of all six ecotypes, were obviously increased under drought stress conditions. On the other hand, drought stress decreased leaf chlorophyll content, photosynthetic rate, stomatal conductance, carboxylation efficiency, and transpiration rate. The results of SDS-PAGE indicated that the abundance of some protein spots was downregulated when the plants were exposed to drought stress, while other protein spots’ abundance was upregulated in such a situation. Under stress conditions, the highest antioxidant enzymatic activity, photosynthetic performance, cell membrane stability, and numbers of protein bands were observed in Baharband and Telvar, while the lowest was related to Fetrezamin. Salicylic acid treatments effectively ameliorated the negative effects of drought stress on Sardari ecotypes through improving the photosynthetic performance, keeping membrane permeability, induction of stress proteins, and enhancing the activity of antioxidant enzymes. The above findings suggest that ecotype ability to maintain photosynthetic performance was important to cope with drought stress.

## 1 Introduction

Drought stress is one of the most devastating environmental stresses, limiting the productivity of crop plants around the world. Drought stress causes a broad range of physiological changes and impairments of metabolic processes, which result in accumulation of reactive oxygen species (ROS) (Abid et al., 2018; Lawlor and Cornic 2002; Qaseem et al., 2019). In response to drought stress, plants activate complex mechanisms, such as the antioxidant defense system, specific proteins like chaperones, and variations in gene expression (Hasan et al., 2018; Huseynova et al., 2007). The mechanisms have been studied for their anti-stress potential in wheat (Hasan et al., 2018), Chinese ryegrass (Li et al., 2019) and, sesame (Najafabadi and Ehsanzadeh, 2017). When antioxidant defense mechanisms are not effective in scavenging and quenching ROS formation, damages to photosynthetic apparatus and cell membrane occur, as well as degradation of biomolecules like pigments and protein, lipid peroxidation, DNA fragmentation, which ultimately result in cell death (Allakhverdiev et al., 2001; Abid et al., 2018; Dalal and Tripathy 2018; Shao et al., 2016). The response of photosynthesis during water-stress has been addressed in recent years, whether the main limitation in photosynthesis is related to stomatal (closure of stomata) or nonstomatal limitations (the decline of mesophyll conductance) and biochemical impairments (Sarabi et al., 2019). It has been shown that there is a significant correlation between the stomatal conductance and photosynthesis response under drought stress, which indicates that stomatal conductance plays a major role in the reduction of leaf photosynthetic rates (Abid et al., 2018; Sarabi et al., 2019).

Salicylic acid (SA), a phytohormone, is a promising compound that can reduce the sensitivity of plants to environmental stresses through regulation of the antioxidant defense system, transpiration rates, stomatal movement, and photosynthetic rate (Nazar et al., 2015). It is evident that SA is a stress-signal molecule that activates abiotic stress-responsive gene expression (Li et al., 2013), and induces the expression of biosynthetic enzymes and proteins in plants under environmental stresses (Nazar et al., 2015; Wang et al., 2019). For example, up-regulation of synthesis of dehydrin-like proteins, chaperone, and heat shock proteins were reported, and also, changes in protein kinase activity, Chlorophyll and rubisco contents were observed (Sun et al., 2009; Kang et al., 2014; Nazar et al., 2015). It is believed that the expression of these genes would lead to reduced ROS production in photosynthetically active tissues (Aldesuquy et al., 2018). Several studies have shown that the application of SA resulted in a positive effect by protecting plants against the oxidative damage caused by drought stress (Kang et al., 2012; Najafabadi and Ehsanzadeh, 2017; Wang et al., 2019; Sankari et al., 2019).

Sardari is one of the most important landraces of common wheat (*Triticum aestivum* L.). The fact that Sardari can grow in various geographical locations attests to its ability to adapt to various abiotic stresses (through both morphological and molecular changes), which is most likely due to a high level of genetic variation (Roostaei et al., 2018). Therefore, elucidating the biochemical and physiological mechanisms of Sardari wheat cultivar under drought stress would help to select cultivars that can adapt to climate change. The present study was carried out to evaluate the physiological and biochemical responses of wheat ecotypes under normal and drought stress conditions and attempts to a better understanding of the effect of SA on drought stress.

## 2 Materials and methods

### 2.1 Plant materials

Seeds from six Sardari wheat ecotypes were obtained from different regions of Kurdistan province (35o57′N, 47o8′E, 1927 m asl) in the west of Iran by the Dryland Agriculture Research Institute, based on their geographical location (Table 1) and differences in spike features like color, size, awn presence, and density.

**Table 1:** Place of collection of ecotypes.

No.	Geographical location name of the ecotype	Elevation above sea level (m)
1	Baharband	2200
2	Kalati	1700
3	Fetrezamin	2000
4	Gavdareh	2050
5	Telvar	1800
6	Tazehabad	2300

#### 2.1.1 Growth conditions

Plants were grown in a greenhouse at the University of Kurdistan in the factorial arrangement based on randomized complete block design with three replications. Six Sardari wheat ecotypes (Baharband, Kalati, Fetrezamin, Gavdareh, Telvar, and Tazehabad) were tested. Plants were treated with salicylic acid at 0.5 mM (controls were untreated) under drought stress conditions (30% of the field capacity). Seeds were planted in plastic pots (four seedlings per pot) and a week after germination the plants were sprayed with salicylic acid. In the drought-stressed treatment, the pots were watered to 30% of FC. Drought stress treatment was carried out before starting the tillering stage until the flowering stage of plants. Measurements were monitored at the anthesis stage and the flag leaves of plants were collected for determination of physiological and biochemical indexes during drought stress.

#### 2.1.2 Lipid peroxidation and membrane permeability

For lipid peroxidation estimation, malondialdehyde (MDA) content was determined using the thiobarbituric acid method as described by Heath and Packer (1968). Membrane permeability was monitored using procedures described by Lutts et al. (1996).

#### 2.1.3 Superoxide dismutase (SOD) assay

Fresh leaf samples (1.0 g) were homogenized in 50mM potassium phosphate buffer (pH = 7), containing 2mM ethylenediaminetetraacetic acid (EDTA) and 1% (w/v) polyvinylpolypirrolidone. The homogenate was centrifuged at 15,000 g, for 10 min at 4 °C (Coban and Göktürk Baydar, 2016). The assay mixture for Superoxide dismutase (SOD) consisted of 835 μL of sodium phosphate buffer 50mM (pH = 8), 33 mL of nitroblue tetrazolium (NBT) 0/75 mM, 33 μL riboflavin, and 33 μL enzyme extract. Absorbance was recorded at 560 nm using a spectrophotometer. One unit enzyme activity was defined as the amount of protein required to inhibit NBT reduction by 50%, which was monitored by absorbance at 560 nm (Alici and Arabaci, 2016).

#### 2.1.4 Catalase (CAT) enzyme activity assay

CAT assay was measured according to Alici and Arabaci (2016) and determined by monitoring the consumption of hydrogen peroxide (H_2_O_2_). Reaction mixture contained 10 μL hydrogen peroxide (15mM) and 50 μL enzyme extract in 3 mL of 50mM potassium phosphate buffer (pH 7). The consumption of H_2_O_2_ was monitored for 1 min at 240 nm following the addition of enzyme extracts to the reaction mixture.

#### 2.1.5 Polyphenol oxidase (PPO) activity assay

PPO assay was performed according to Chance and Maehly (1955) through monitoring H_2_O_2_ consumption at 420 nm. Reaction mixture contained 0.2M potassium phosphate buffer (pH 7.6), 0.02M pyrogallol and 100 μL of enzyme extract. Changes in the absorbance at 420 nm were recorded for 1 min. One PPO unit is defined as one mmol pyrogallol oxidized per gram fresh weight per min.

#### 2.1.6 Protein analysis

Protein concentration in enzyme extracts was determined using Bradford's method (1976). Soluble proteins from leaves were separated on a 12% SDS-PAGE according to Laemmli (1970) method using a Mini Protean II Dual Slab Cell (Bio-Rad). Protein gels were stained with Coomassie brilliant blue R-250 solution.

### 2.2 Sugars content

The sucrose, fructose, glucose concentration in leaves was determined by spectrophotometry using the protocol outlined by Wu et al. (2015).

#### 2.2.1 Physiological parameter

Net photosynthesis (Pn), stomatal conductance (gs), internal CO_2_ concentration (Ci), and transpiration rate (E) were determined at the flowering stage using a portable gas exchange measuring system (Li 6400, Li-Cor, USA). Carboxylation efficiency (MC) was calculated by dividing Pn by Ci (Fischer et al., 1998), photosynthetic water use efficiency (PWUE) was calculated by dividing Pn by gs. Measurements were performed between 10:00 am and 12:00 noon under atmospheric CO_2_. Chlorophyll content extracted in 80% acetone solution. Extracts were measured spectrophotometrically at 663nm and 645nm. Chlorophyll *a* and *b* concentrations were calculated according to Arnon (1949).

### 2.3 Statistical analysis

Data were analyzed using SAS (9.2) statistical program and means were compared using an LSD (Least Significant Difference) test (*P* < 0.05). Also, to show a possible difference between treatments, the percentage difference between the two data was calculated by dividing the difference between the first value and the second value by the second value. Principal component analysis (PCA) and Heatmap analysis were performed by R language. All parameters and ecotypes were included in the analysis.

## 3 Results

### 3.1 Drought stress increased membrane electrolyte leakage

**Figure 1:**
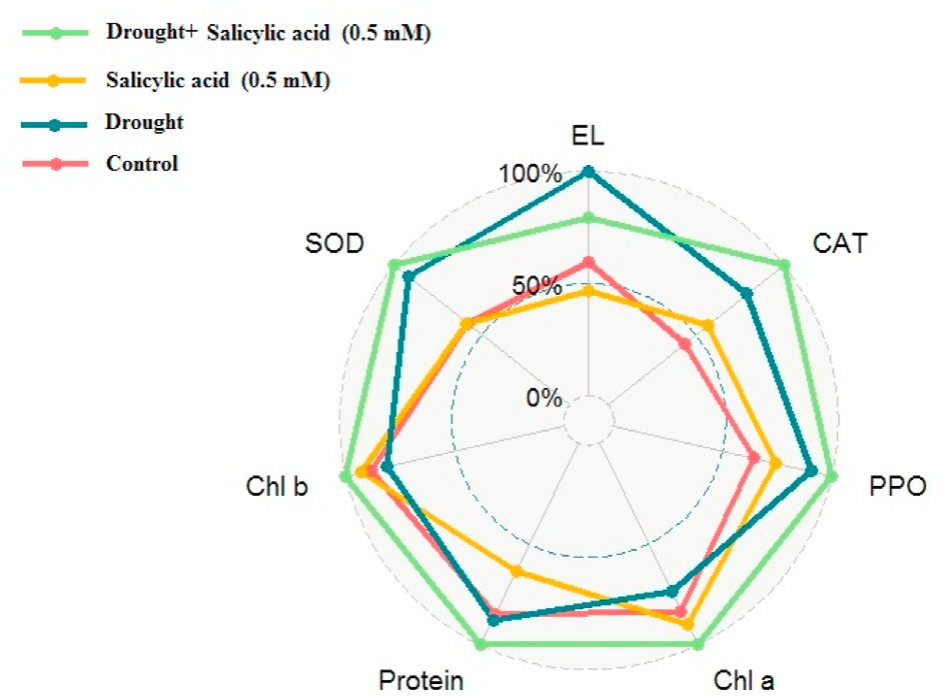
Effect of salicylic acid foliar spray on drought stress and non-stressed plants on catalase (CAT), polyphenol oxidase (PPO), superoxide dismutase (SOD), protein, chlorophyll a (Chl a), chlorophyll b (Chl b), and membrane electrolyte leakage (EL) traits.

Membrane electrolyte leakage of all six ecotypes was increased under drought stress (Table 2). Compared to the control the most impacted cultivars are Fetrezamin and Gavdareh, with 67.88% and 63.6% higher, respectively. As expected, the results showed that exogenous SA application significantly reduced the membrane electrolyte under non-stress and stress conditions by 21.63% and 20.79%, respectively, compared to the control (Figure 1).

### 3.2 Drought stress significantly enhanced SOD, CAT and PPO activities

In plants that were not treated with SA, drought stress significantly enhanced the SOD activity of leave tissues compared to the control (Table 2). However, under drought stress, these increases were more noticeable in the Baharband cultivar than in the other ecotypes (42.57%, Table 2). In drought-stressed plants, the lowest SOD activity was observed in Fetrezamin and Gavdareh cultivars, by 20.8 (unit mg protein^−1^) and 20.9 (unit mg protein^−1^), respectively (Table 2). In addition, the increase in SOD activity was consistently accompanied with a significant increase in CAT activity in the leaves of drought-treated ecotypes compared to the control, with Gavdareh cultivar had the lowest value for CAT activity and Baharband cultivar had the highest value. Under drought stress, PPO enzymatic activity increased, and in both control and stress conditions, Fetrezamin showed the lowest PPO activity (0.6 unit mg protein^−1^), while the highest increase was observed in Telvar and Baharband (0.83 unit mg protein^−1^) (Table 2).

**Table 2:** Effect of drought stress on Sardari wheat ecotypes.

Treatment	Ecotype	Membrane electrolyte leakage %	Protein (μMg^−1^FW)	Superoxide dismutase (unit mg protein^−1^)	Catalase (unit mg protein^−1^)	Net photosynthesis (μmol co_2_ m^−2^ s^−1^)	Stomatal conductance (mmol (H_2_O) m^−2^ s^−1^)	Internal CO_2_ concentration (μmolmol^−1^)	Transpiration rate (m^−2^s^−1^ mmol)	Polyphenol oxidase (unit mg protein^−1^)
Control	Kalati	42.74 ± 3.98^f^	3.59 ± 0.49 ^bc^	13.91 ± 0.58 ^fg^	0.26 ± 0.02 ^def^	8.33 ± 0.54^b^	0.64 ± 0.02^a^	391.6 ± 1.33 ^ef^	6.76 ± 0.38^b^	0.45 ± 0.04^e^
	Tazehabad	42.2 ± 1.88^f^	2.79 ± 0.37^d^	14.17 ± 0.30 ^efg^	0.26 ± 0.02 ^def^	8.58 ± 0.88^b^	0.73 ± 0.06^a^	393.78 ± 0.68 ^de^	7.23 ± 0.28^b^	0.48 ± 0.04^e^
	Fetrezamin	49.42 ± 2.39^e^	2.3 ± 0.18^e^	13.08 ± 0.28^g^	0.25 ± 0.02^ef^	5 ± 0.64^c^	0.46 ± 0.06^b^	392.53 ± 2.66^e^	6.17 ± 0.44^b^	0.36 ± 0.03^f^
	Gavdareh	51.15 ± 2.39 ^de^	2.4 ± 0.06 ^de^	13.35 ± 0.36^g^	0.22 ± 0.01 ^ef^	5.03 ± 0.55^c^	0.5 ± 0.07^b^	394.28 ± 1.60 ^de^	6.26 ± 0.39^b^	0.38 ± 0.02^f^
	Baharband	27.85 ± 4.23^h^	3.31 ± 0.16^c^	15.61 ± 0.16 ^ef^	0.29 ± 0.02 ^de^	9.87 ± 0.81^a^	0.7 ± 0.02^a^	386.33 ± 2.80^g^	8.41 ± 0.78^a^	0.56 ± 0.05^d^
	Telvar	35.73 ± 2.45^g^	3.38 ± 0.21^c^	15.85 ± 0.44^e^	0.31 ± 0.02^d^	9.85 ± 0.74^a^	0.75 ± 0.05^a^	387.55 ± 1.46 ^fg^	8.96 ± 0.76^a^	0.59 ± 0.04 ^cd^
Drought	Kalati	67.02 ± 3.34^b^	3.71 ± 0.12 ^abc^	22.93 ± 1.07^c^	0.48 ± 0.04^b^	3.12 ± 0.46^d^	0.16 ± 0.01 ^cd^	403.01 ± 2.32^c^	1.25 ± 0.11^c^	0.74 ± 0.04^b^
	Tazehabad	71.92 ± 3.42^b^	3.84 ± 0.17 ^ab^	23.64 ± 0.53^c^	0.5 ± 0.04 ^ab^	2.8 ± 0.63^d^	0.16 ± 0.01 ^cd^	403.45 ± 2.86^c^	1.13 ± 0.20^c^	0.76 ± 0.06^b^
	Fetrezamin	82.97 ± 4.40^a^	3.44 ± 0.17 ^bc^	20.8 ± 1.17^d^	0.39 ± 0.03^c^	1.5 ± 0.20^f^	0.11 ± 0.02^d^	411.26 ± 1.89^b^	0.85 ± 0.19^c^	0.60 ± 0.04 ^cd^
	Gavdareh	83.70 ± 4.82^a^	3.42 ± 0.16^c^	20.9 ± 1.26^d^	0.38 ± 0.03^c^	1.73 ± 0.17 ^ef^	0.16 ± 0.05 ^cd^	418.5 ± 1.79^a^	0.91 ± 0.18^c^	0.64 ± 0.04^b^
	Baharband	58.93 ± 4.92^c^	4.06 ± 0.17^a^	25.83 ± 0.56^b^	0.56 ± 0.04^a^	4.82 ± 0.35^c^	0.23 ± 0.04^c^	397.5 ± 1.83^d^	1.6 ± 0.16^c^	0.83 ± 0.04^a^
	Telvar	56.79 ± 3.93 ^cd^	4.04 ± 0.22^a^	27.6 ± 0.20^a^	0.54 ± 0.04^a^	5.17 ± 0.25^c^	0.25 ± 0.05^c^	386.55 ± 4.05^g^	1.61 ± 0.12^c^	0.83 ± 0.03^a^

In each column different letters (a–f) mean significant differences at *P* ≤ 0.05. Means ± S.D from the three experiments.

Under water deficit stress, the SA application increased antioxidant enzymatic activity compared to controlled plants. As indicated in Figure 1, although SA application to non-stressed plants showed more SOD activity, this increase was not significant. However, exogenous application of SA under drought stress resulted in an increase of SOD activity (42.16% more compared to control plants) (Figure 1). Remarkably, SA application increased CAT activity regardless of growth conditions (watered plants and drought-stressed plants), the higher increase was observed in drought-stressed plants (56.6% more compared to watered plants) (Figure 1). In addition, foliar application of SA significantly to drought-stressed plants enhanced PPO activity by 18.18% compared with control plants (SA-untreated plants) (Figure 1).

### 3.3 Stomatal conductance and photosynthesis are reduced under drought stress

These results show that, regardless of the ecotypes and growth condition, water stress reduced the gs in all plants (Table 2). Fetrezamin cultivar had the lowest gs values in both controlled and drought-stressed plants, with 0.46 and 0.11 (mmol (H_2_O) m^−2^ s^−1^), respectively (Table 2); whereas, the Telvar cultivar showed the highest gs values under both control and drought conditions (Table 2). Furthermore, the reduction in gs was accompanied by a decline in photosynthesis. In drought stress condition, most and least values of Pn was observed in Telvar and Fetrezamin cultivars, respectively (Table 2).

While transpiration value considerably decreased in plants exposed to drought stress, water use efficiency (PWUE) increased in the same condition. Under drought stress condition, Telvar showed the greatest transpiration rate compared to the other ecotypes; however, this difference was not significant (Table 3). Drought stress increased the internal CO_2_ concentration (Ci) in all ecotypes. Interestingly, Telvar was the only ecotype which showed a decline in Ci under drought stress (Table 2).

**Table 3:** Effect of salicylic acid foliar spray on drought stress and non-stressed plants on photosynthetic parameters and catalase.

Treatment	SA (mM)	Net photosynthesis (μmol co_2_ m^−2^ s^−1^)	Stomatal conductance (mmol (H_2_O) m^−2^ s^−1^)	Internal CO_2_ concentration (μmolmol^−1^)	Transpiration rate (m^−2^s^−1^ mmol)	photosynthetic water use efficiency (mol^−1^ μmol co_2_)	Carboxylation efficiency (m^−2^s^−1^ mmol)
Control	0	6.81 ± 0.44^b^	0.63 ± 0.03^a^	393.46 ± 0.88^c^	6.76 ± 0.88^b^	1.07 ± 0.30^d^	0.017 ± 0.88^b^
	0.5	8.75 ± 0.69^a^	0.63 ± 0.04^a^	388.56 ± 0.88^d^	7.84 ± 0.88^a^	1.18 ± 0.48^c^	0.023 ± 0.88^a^
Drought	0	2.81 ± 0.37^d^	0.12 ± 0.01^c^	407.91 ± 0.88^a^	1.13 ± 0.88^c^	2.48 ± 0.195^b^	0.006 ± 0.88^c^
	0.5	3.57 ± 0.41^c^	0.23 ± 0.02^b^	398.84 ± 0.88^b^	1.32 ± 0.88^c^	2.70 ± 0.254^a^	0.007 ± 0.88^c^

In each column different letters (a–f) mean significant differences at *P* ≤ 0.05. Means ± S.D from the three experiments.

In this study, SA application significantly improved the ability of plants to alleviate the adverse effects of drought, particularly improved the Pn, gs, and Ci values (Table 3). The most positive impact was observed on gs values, with 91.6% compared to SA-untreated plants (Table 3). However, there was no difference in the stomatal behavior among the non-stressed plants (control and SA treated plants). Exogenously applied SA improved photosynthesis in both control and drought-stressed plants by 28.48% and 25%, respectively, compared to untreated plants (Table 3). SA foliar spray significantly increased carboxylation efficiency (MC) and transpiration (E) in non-stressed plants (35.29% and 15.97% higher compared to control plants) (Table 3), while, this increase was not significant in drought-stressed plants (Table 3). As expected, the SA application significantly increased PWUE in both control and drought-stressed plants. The highest PWUE was related to stressed-plants treated with SA (2.70 mol^−1^ μmol co_2_) (Table 3).

**Figure 2:**
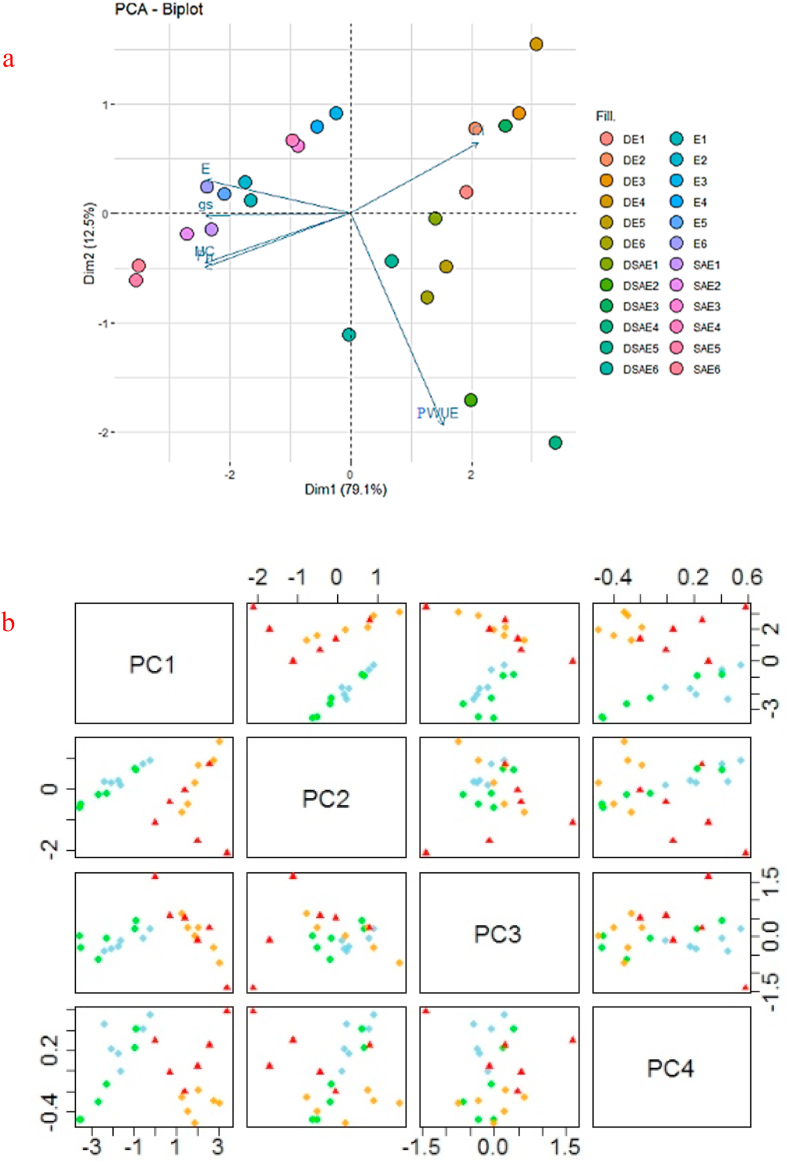
Biplot of principle component analysis (a) for the first two principle components of photosynthetic parameters, ecotypes, drought stress and salicylic acid under non-stress and drought stress treatments. The score plot (b) for the four treatments; control, drought stress, non-stress, salicylic acid and drought stress∗salicylic acid were indicated in blue, green, orange and red, respectively. Abbreviations: Ci, internal-stomatal CO_2_ concentration; PWUE, photosynthetic water use efficiency; MC, Carboxylation efficiency; Pn, net photosynthesis rate; gs, stomatal conductance; D, drought stress; SA, salicylic acid; E, respiration rate D, drought stress; SA, salicylic acid; DSA, drought stress∗salicylic acid; DSAE, drought stress∗salicylic acid∗Ecotype. Ecotypes: Kalati (E1), Tazehabad (E2), Fetrezamin (E3), Gavdareh (E4), Baharband (E5) and Telvar (E6).

**Figure 3:**
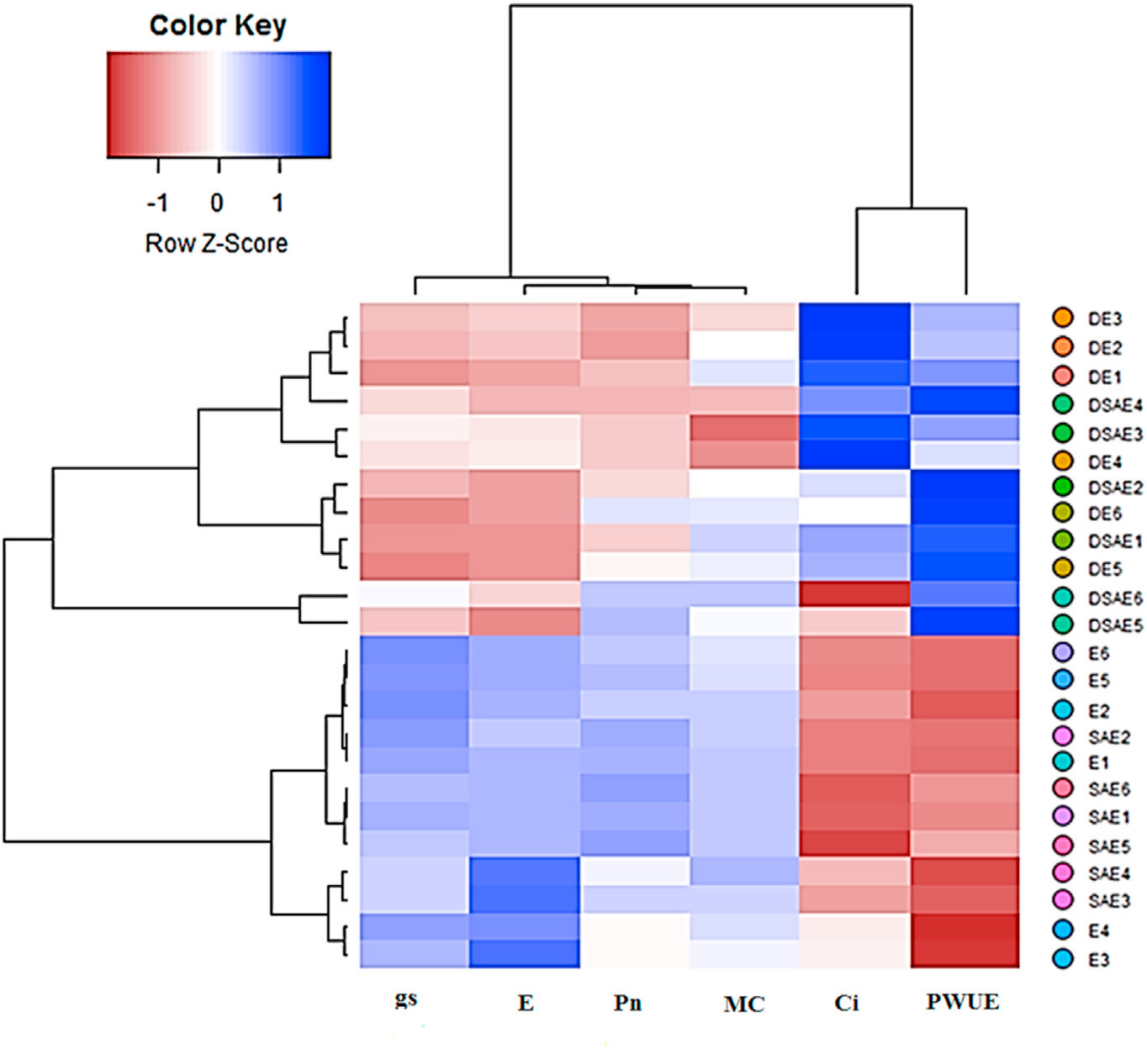
Heatmap of photosynthetic parameters across wheat ecotypes and treatments. Rows represent 6 wheat ecotypes under various treatments (drought stress and salicylic acid) and columns represent the photosynthetic parameters. The color key, from-blue-to-red color represents the value of photosynthetic parameters from low to high. Abbreviations: Ci, internal-stomatal CO_2_ concentration; E, respiration rate; PWUE, photosynthetic water use efficiency; MC, Carboxylation efficiency; Pn, net photosynthesis rate; gs, stomatal conductance; D, drought stress; SA, salicylic acid; DSA, drought stress∗salicylic acid; DSAE, drought stress∗salicylic acid∗Ecotype. Ecotypes: Kalati (E1), Tazehabad (E2), Fetrezamin (E3), Gavdareh (E4), Baharband (E5) and Telvar (E6).

The heatmap and principal component analysis were conducted on the data of the six-wheat ecotype × six traits selected of leaf photosynthetic parameters, and the association between the traits was compared across the control, SA, and drought stress treatments (Figures 2 and 3). Photosynthetic changes were observed in all ecotypes after the application of drought stress, which resulted in five groupings of ecotypes, and the traits were grouped into three clusters with the strongest differentiating powers at Ci (Figures 2 and 3). Interestingly, controlled plants (SA-treated and untreated plans) and drought-stressed plants (SA-treated and untreated plans) were separated from each other by two opposing groups (Figures 2 and 3). This separation of samples between two conditions indicates different photosynthetic levels in the wheat flag leaves of 6 ecotypes under drought stress. One group (controlled plants) contained the Pn, gs, Mc, and evaporation, whereas the other group was most strongly influenced by Ci and PWUE.

PCA findings showed a tight positive association between Pn and MC while they were associated negatively with intracellular CO_2_ concentration. This type of specific association was the most pronounced in E5 and E6 (both SA-treated and SA + stressed plants) (Figures 2 and 3), which indicates the up-regulation of polypeptides (Figure 6c and Figure S3 and 7), high mean values for antioxidant enzymes activity, and lowest damage of chloroplast and chlorophyll pigments (Table 2); however, the opposite was true of E3 (stressed plants), which gave the lowest Pn regardless of SA-treatment. The gs in E3 was the most sensitive parameters to drought stress, leading to a severely increased internal-stomatal CO_2_ concentration, cell membrane electrolytic leakage, MDA concentration (Figure 5), and down-regulation of polypeptides (Figure 6b and Figure S2 and 7), as a result of which this group became the most sensitive at drought stress. The drought sensitivity of the photosynthetic performance of E1 and E2 was intermediate.

### 3.4 Drought stress reduced chlorophyll content

Drought stress led to a remarkable decrease in chlorophyll a and b compared to the control (26.22% and 18.66%, respectively compared to un-treated plants) (Figure 1). As it can be seen in Figure 1, foliar application of SA significantly enhanced chlorophyll a and b (7.05% and 4.08%, respectively compared to controlled plants). There was a significant difference in chlorophyll content between all six ecotypes, as Baharband and Fetrezamin ecotypes had the highest and lowest amounts, respectively (Table 4).

**Table 4:** Main effect of Sardari wheat ecotypes on chlorophyll a and b, and Carboxylation efficiency.

Ecotypes	Carboxylation efficiency (m^−2^s^−1^ mmol)	Chlorophyll a (μg/ml)	Chlorophyll b (μg/ml)
Kalati	0.015^a^	5.26 ^ab^	4.84 ^ab^
Tazehabad	0.015^a^	5.39 ^cd^	4.77 ^ab^
Fetrezamin	0.0066^b^	4.83^c^	4.33^c^
Gavdareh	0.0083^b^	4.87^c^	4.53 ^bc^
Baharband	0.017^a^	5.82^a^	5.03^a^
Telvar	0.018^a^	5.81^a^	5.07^a^

In each column different letters (a–f) mean significant differences at P ≤ 0.05.

### 3.5 Sugars content

**Figure 4:**
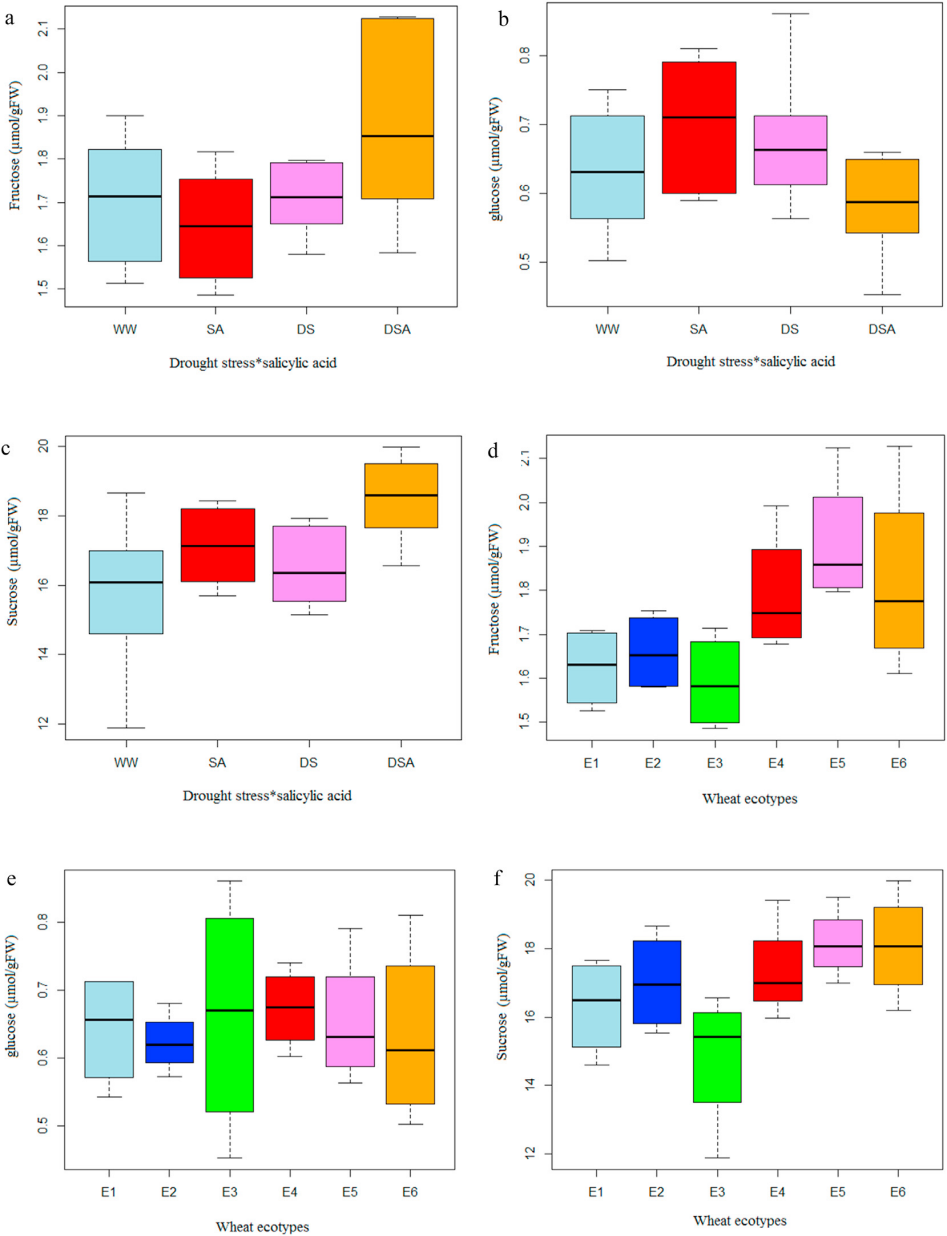
Box Plots of sugar Levels that showed different treatments, drought stress and salicylic acid (a, b, c) and ecotypes (d, e, f), are color-coded. Ecotypes: Kalati (E1), Tazehabad (E2), Fetrezamin (E3), Gavdareh (E4), Baharband (E5) and Telvar (E6); well warered (ww); salicylic acid (SA); drought stress (DS); drought stress∗salicylic acid (DSA).

The effect of drought stress on sugar content is shown in Figure 4. The results showed that drought stress significantly enhanced the sucrose content compared to the control (Figure 4c). Sucrose content was significantly higher in plants treated with SA compared to the control plants. This difference was even greater when the SA-treated plants were exposed to drought stress, as the highest sucrose content was observed in drought stressed-plants treated with SA.

As indicated in Figure 4f, the highest and lowest value for sucrose content were obtained in E6 ecotype and E3 ecotype, respectively. Fructose content was decreased following drought stress and SA treatments (Figure 4a), while, a highly significant increase was observed in the level of fructose in the SA-treated plants when they exposed to drought stress (Figure 4a). Fructose content considerably varied between all six ecotypes, as the highest and lowest content were recorded in E5 and E3 ecotypes, respectively (Figure 4d). Glucose content was also significantly affected by the SA treatment and ecotype (Figure 4). The lowest and highest glucose content was observed in SA-treated plants with and without drought stress, respectively (Figure 4b). The E3 cultivar showed a significantly higher Glucose content in comparison to other ecotypes, while the lowest sucrose and fructose contents were recorded for the E3 ecotype (Figure 4e).

### 3.6 Drought stress increases lipid peroxidation

**Figure 5:**
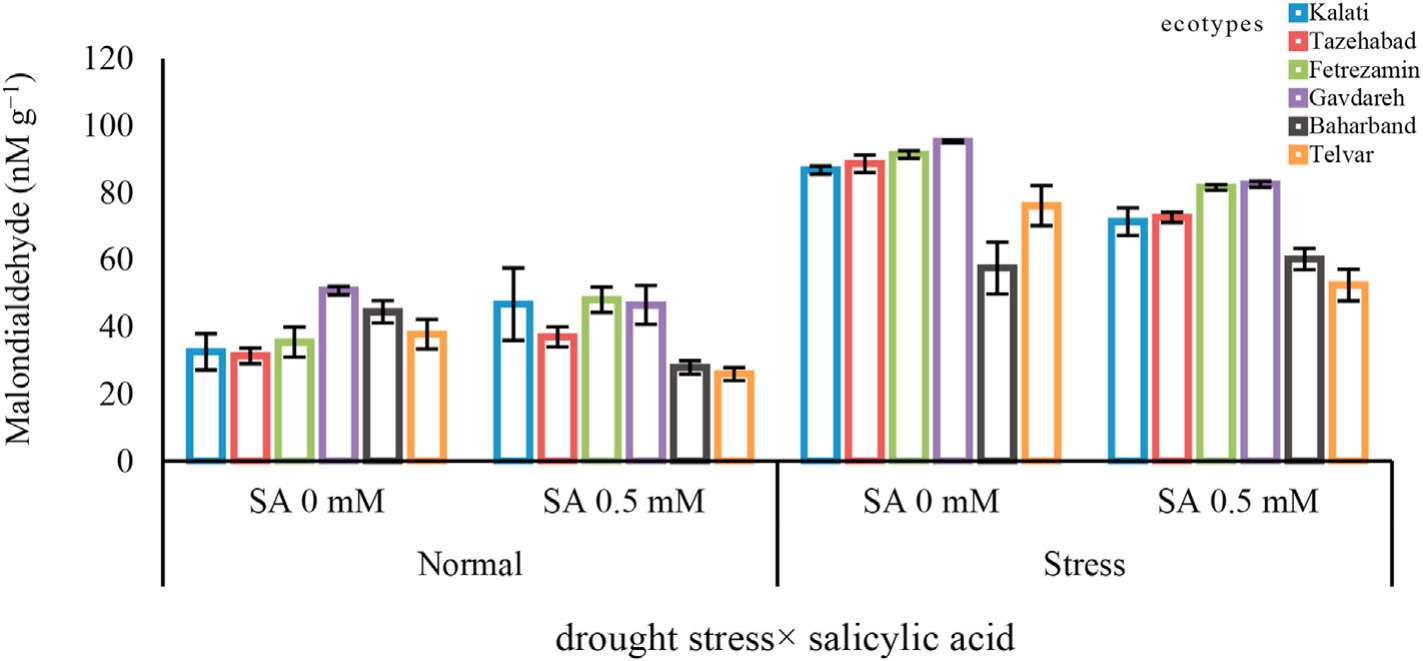
Interaction between drought stress and salicylic acid on malondialdehyde (MDA) in Sardari wheat ecotypes. In each figure, means with the same letter are significantly different according to LSD test at P < 0.05. Means ± S.D from the three experiments.

The investigation into lipid peroxidation revealed that there was a significantly increased lipid peroxidation for all the six ecotypes under drought stress conditions compared to controlled plants (Figure 5). Under drought stress, the Gavdareh ecotype showed the highest levels of lipid peroxidation compared to other ecotypes (Figure 5). As it can be seen from Figure 5, the Baharband ecotype showed the lowest lipid peroxidation under drought conditions without SA. application. Data in Figure 5 also showed that lipid peroxidation was suppressed by the SA. treatment under drought stress. The highest malondialdehyde was observed in Gavdareh (SA-untreated), while, Telvar (SA-treated) had the lowest malondialdehyde.

### 3.7 Protein content and composition are increased under drought stress

The results also show that protein contents increased when plants were subjected to drought stress (Table 2). Under both controlled and stress conditions, the highest protein contents were obtained from Baharband and Telvar, while the lowest protein contents were observed in Fetrezamin ecotypes (Table 2). SA application to plants under drought stress remarkedly increased soluble protein content compared to the plants only treated with drought stress (Figure 1).

**Figure 6:**
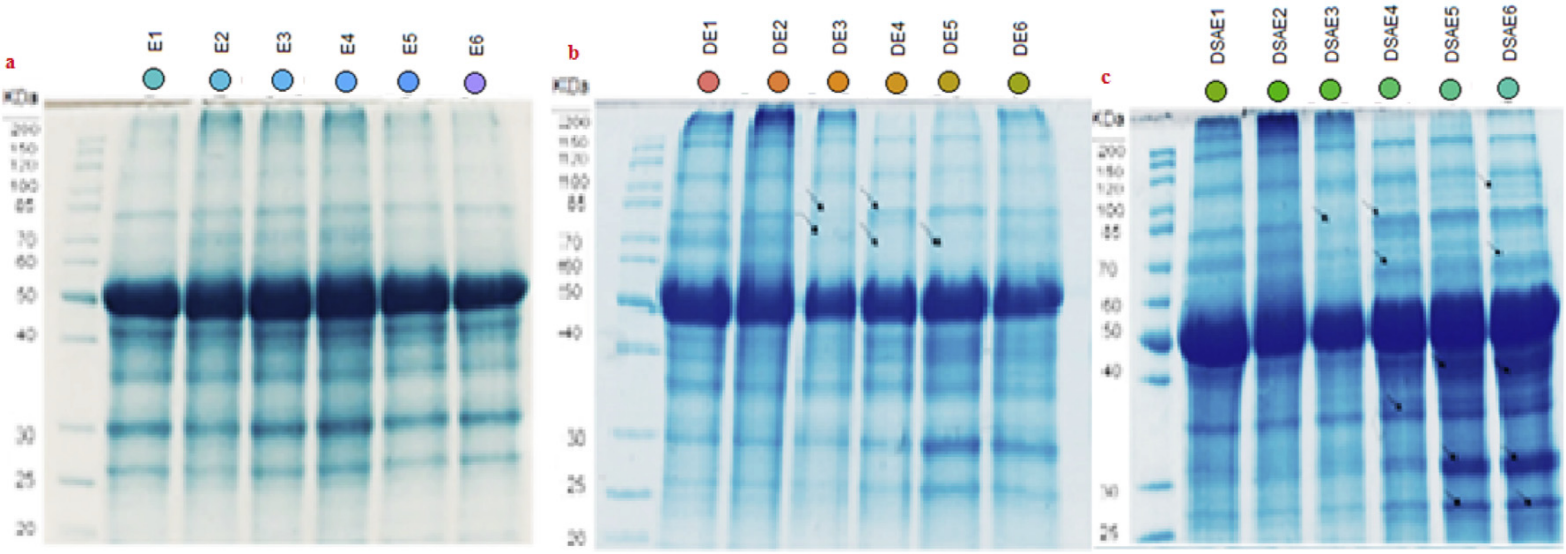
SDS PAGE for ecotypes (numbered 1 to 6) Kalati (E1), Tazehabad (E2), Fetrezamin (E3), Gavdareh (E4), Baharband (E5) and Telvar (E6). Plants were grown under watered condition (a), drought stress condition (b) and drought stressed plants treated with salicylic acid (c). Abbreviations: salicylic acid; DSA, drought stress∗salicylic acid; DSAE, drought stress∗salicylic acid∗Ecotype.

Under drought stress, polypeptides were either downregulated or upregulated (Figure 6b and Figure S2). The major changes in profiles of protein bands were observed in Fetrezamin ecotype; namely, a decrease in the intensity of bands at 40kDa, 70kDa, and 70–85kDa in drought-stressed plants compared to the control. Interestingly, the application of exogenous SA increased the intensity of polypeptides in all tested ecotypes. Specifically, bands at 15kDa, 16kDa, 25kDa, 36kDa, 42kDa, 43kDa, 45kDa, 47−49kDa, 52kDa, 53kDa, 59kDa, 71kDa, 73kDa, 76−80kDa, and 82kDa were significantly upregulated in some ecotypes (Gavdareh, Baharband, and Telvar) compared to the control (Figure 6c and Figure S3).

**Figure 7:**
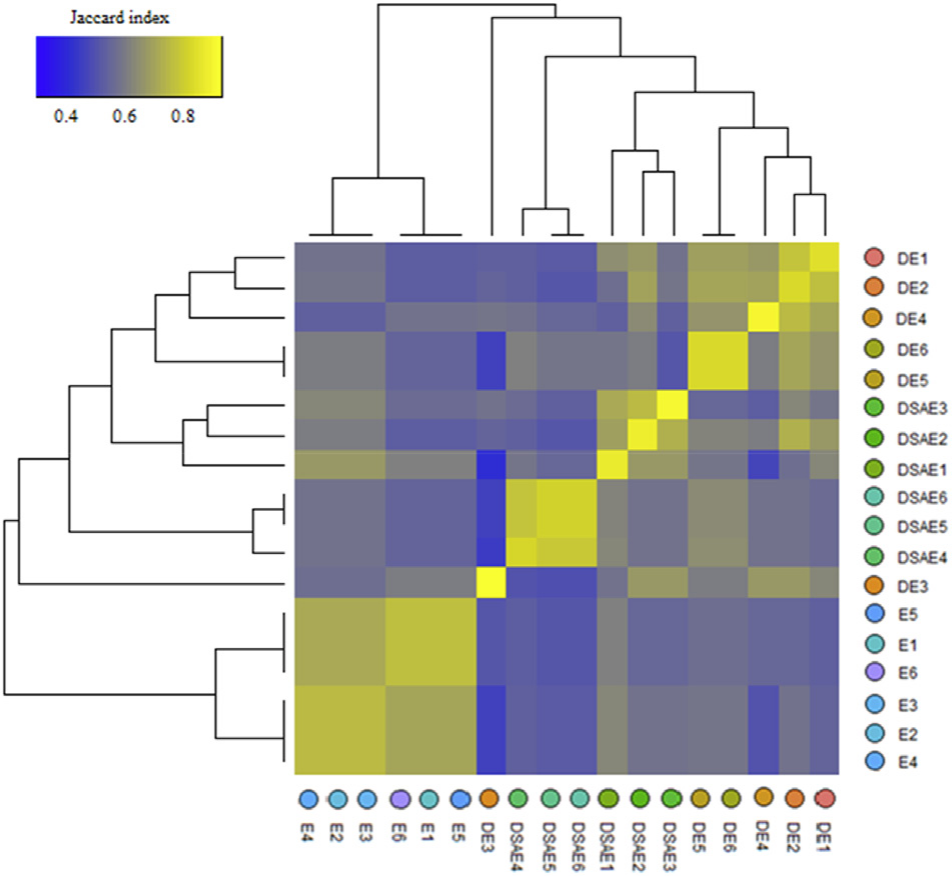
Heatmap was calculated using the Jaccard index for ecotypes. Jaccard-indices comparing the similarity in the sets of bands regulated by SDS PAGE in each of 6 ecotypes. Abbreviations: D, drought stress; SA, salicylic acid; DSA, drought stress∗salicylic acid; DSAE, drought stress∗salicylic acid∗Ecotype. Ecotypes: Kalati (E1), Tazehabad (E2), Fetrezamin (E3), Gavdareh (E4), Baharband (E5) and Telvar (E6).

Heatmap and Clustering for Jaccard-indices generated from protein bands counts were grouped into six clusters (Figure 7). In this way, it became evident that differences existed in the drought stress sensitivity of 6 ecotypes. Cluster I was characterized by control plants. Interestingly, only Fetrezamin ecotype (drought-treated plants) was classified into Cluster II; it was the ecotype with the lowest protein bands under drought conditions. Based on the heatmap of protein bands, Clusters 3 (SA-treated) and 5 proved to be the most drought tolerant Clusters. It is noteworthy that the three ecotypes (SA-treated forming cluster III) had high values of polypeptides under drought conditions, whereas Clusters 4 (SA-treated) and Clusters 6 (SA-untreated) were intermediate in their reactions.

## 4 Discussion

Drought stress-induced ROS production due to a reduction in light absorption and photosynthetic electron transport, which induced photo-oxidative damages to photosystems (Dalal and Tripathy 2018; Yudina et al., 2020; Hu et al., 2018). As a result of these damages, a decline in Pn happens and peroxidation in the cell membrane is enhanced. The results suggest that drought stress did cause damages to the membrane system in Sardari cultivar, which increased lipid peroxidation and plasma membrane electrolytic leakage (Table 2). There is a positive correlation (98%, *P* < 0.05) between ionic leakage and lipid peroxidation. Disorganization of the chloroplast membrane system, due to damages and swelling of thylakoid membranes (both stromal and granal) has also been reported on maize and wheat under drought stress condition (Tian et al., 2013; Shao et al., 2016). A number of studies have demonstrated that exogenously applied SA maintained the integrity of chloroplast and thylakoid structures under drought stress conditions (Aldesuquy et al., 2018). In agreement with these previous reports, this result indicated that exogenous SA application improved cell membrane stability, decreased MDA concentration, and limited electrical conductivity in leaves of all wheat ecotypes in both control and drought stress conditions (Figures 1 and 5).

Antioxidant gene expression could be responsible for the increase in SOD and CAT activities in some of the wheat ecotypes under drought stress condition. These enzyme activities help with detoxification and reduce oxidative damage to cells through converting harmful ROS into H_2_O_2_ and less toxic compounds (Abid et al., 2018). In addition, there is a relationship between CAT and MDA in various wheat ecotypes, which is in agreement with previous studies (Hasan et al., 2018; Abid et al., 2018; Khalvandi et al., 2019; Li et al., 2019). SA exerts anti-stress effects by inducing the expression of antioxidant biosynthetic genes (antioxidant potential) and elevating the activity of antioxidant enzymes (Li et al., 2013; Najafabadi, and Ehsanzadeh, 2017; Wang et al., 2019). In the current study, lower electrolyte leakage in SA-treated plants can be attributed to the protection of cell membrane structure from oxidation, which suggests an improved performance of ROS scavenger activity, such as CAT, PPO, and SOD activities.

The results of this study showed that drought induced excessive production of ROS in all six ecotypes. This phenomenon caused damages to photosynthetic apparatus as evidenced by a significant increase in transpiration, stomatal and carboxylation efficiency, photosynthesis, internal-stomatal CO_2_ concentration, and photosynthetic water use efficiency (Table 2). However, the photosynthetic responses of wheat ecotypes to drought stress appears to be variable (Table 2). The reason could be attributed to the inhibitory effect of drought on the stomatal (stomatal closure) and non-stomatal (impairments of metabolic processes) factors (Sarabi et al., 2019). One important response of plants to drought stress is to limit the stomatal opening to prevent water loss via transpiration; consequently, the diffusion of CO_2_ into the leaf is restricted, which may induce the reduction of photosynthesis (Shao et al., 2018; Zhu et al., 2020; Dawood and Abeed, 2020). In all six ecotypes in this study, carboxylation efficiency limitation accompanied by notable decreases in photosynthesis. The phenomenon that could indicate stomatal limitation, lower carbon fixation, as also, damages of chloroplast constituents such as chlorophyll and lipids which is consistent with reports by Nazar et al. (2015). These alterations in mesophyll conductance along with a reduction in the supply of CO_2_ to rubisco may be linked to physical alterations in the structure of the intercellular spaces due to leaf shrinkage, or to alterations in any biochemical reactions or changes in the composition of membrane at drought stress (Lawlor and Cornic 2002; Grigorova et al., 2012). Clearly, thylakoid membrane lipid skeleton and pigment-protein complexes are critical for the maintenance of photosystem II (PS II) activity under drought stress (Tian et al., 2013; Hasan et al., 2018). The results showed an increase in photosynthetically water use efficiency (Table 3). It is well documented that there is a direct correlation between water use efficiency and a simultaneous decline in gs and lower transpiration rate in plants under water shortage. An increment in PWUE during drought stress has been previously reported in many plants such as rice (Karaba et al., 2007), winter wheat (Xue et al., 2006), and chickpea cultivars (Mafakheri et al., 2010). Nevertheless, exogenous SA application mitigated the inhibitory effect of drought stress on the photosynthetic capability of Sardari ecotypes (Table 3). Similar observations were reported about improving photosynthetic rate under drought stress (Shao et al., 2018; Nazar et al., 2015) and cadmium stress (Wang et al., 2019). SA can help improve the availability of carbon for photosynthesis by increasing the stomatal opening. SA may also regulate certain metabolic factors associated with carbon uptake and/or fixation in the chloroplast. Moreover, SA could relate to rubisco concentration and activity; it also helps to keep the integrity of light-harvesting apparatus (Nazar et al., 2015), a mechanism which could be responsible for increasing photosynthesis under drought stress.

**Figure 8:**
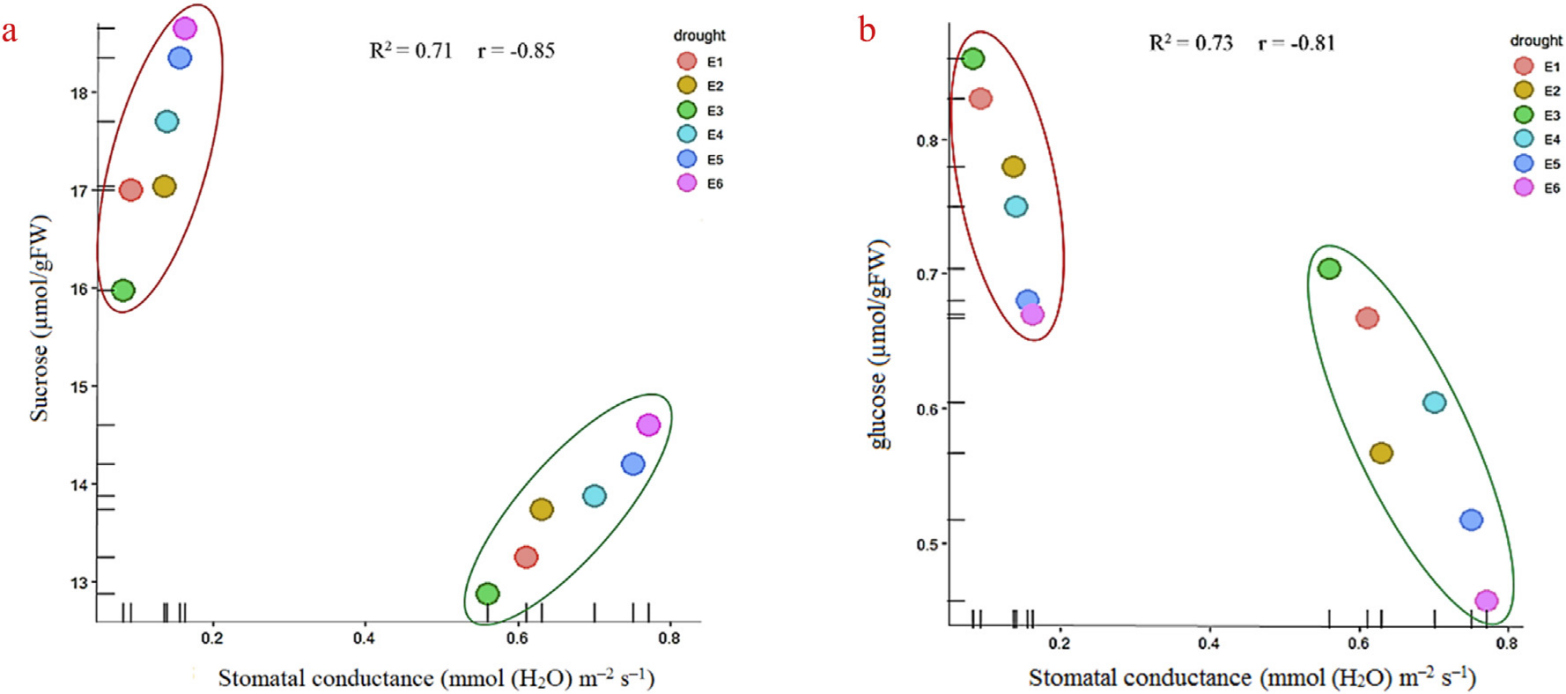
Corrolation analysis. Circle green color represents well-watered samples; circle red color represents drought-stressed samples. Relation of leaf stomatal conduction and abundance of sucrose (a) and glucose (b). Abbreviations: Kalati (E1), Tazehabad (E2), Fetrezamin (E3), Gavdareh (E4), Baharband (E5) and Telvar (E6).

An interesting aspect of our study was to combine knowledge from physiological parameter (gs) of leaves in wheat ecotypes with the sugar response under drought stress (Figure 8b). The results revealed that there was an inverse relationship between gs and sucrose in all ecotypes. Under drought stress conditions, sugar production might exceed the plant's phloem-loading and translocation capacity; as a result, sugar accumulates in leaves. Sucrose, or the products of sucrose cleavage (glucose and fructose) might be carried toward the guard cells via sucrose and hexose transporters. The mechanism that leads to stomatal closure and reduces water loss (Kottapalli et al., 2018). In sensitive ecotypes, the sensing of high sugar levels within guard cells leads to enhanced (and perhaps faster) closure of the stomata. Furthermore, sugar accumulation in plant organs (mainly sucrose) are important osmoprotectants and energy sources of plant cells under abiotic stress, which can induce plant tolerance to abiotic stress. Previous studies showed that a higher accumulation of sucrose might be involved in the regulations of carbohydrate metabolism, sugar metabolism, and sugar transport (Du et al., 2020).

Accumulation of protein stress is necessary for maintaining osmotic balance, and membrane stability under stressed environment (Qaseem et al., 2019). Under drought stress, soluble protein content and expression of polypeptides were variable among the studied wheat ecotypes (Table 2; Figure 6). In ecotypes Fetrezamin and Gavdareh, the expression of polypeptides at 40kDa, 70kDa, and 70–85kDa were down-regulated under drought stress (Figure 6). Earlier proteomic analysis of rice seedlings and barley leaf under water deficit revealed alterations in the levels of proteins involved in electron transport (reduction in pigment-protein complexes) energy balance, transcription, metabolism, chaperons, and protein synthesis (Ashoub et al., 2013; Dalal and Tripathy 2018). It is well documented that drought stress reduces the photosynthetic efficiency through oxidative damages to lipids, nucleic acids, and proteins (Tian et al., 2013; Hasan et al., 2018), which may result in down-regulation of photosynthetic proteins of PSII, PSI, and light-harvesting Chl-proteins, chaperones and chlorophyll a-b binding proteins (Dalal and Tripathy 2018; Hasan et al., 2018; De Oliveira et al., 2019). Several reports have suggested that a good correlation could be existed between leaf protein patterns and preservation of integrity and internal structure of photosynthetic organs such as chloroplasts and chloroplast components (Tian et al., 2013) and also higher activities of chloroplastic antioxidant enzymes (Sankari et al., 2019).

It has also reported that exogenous SA application can has an important regulatory role in protein synthesis associated with a systemic stress response. In some ecotypes (Gavdareh, Baharband, and Telvar) in our study, the application of exogenous SA remarkably increased the expression and intensity of certain polypeptides. In other studies, analysis of protein expression patterns revealed that 35 key proteins in the metabolic processes were induced by SA; proteins which are associated with physiological functions, including signal transduction, photosynthesis, carbohydrate metabolism, energy production, protein metabolism, and stress defense (Kang et al., 2012). In this experiment, a high abundance of polypeptides with molecular masses of ~25–50kDa, was observed in the SA-treated plants (Gavdareh, Baharband, and Telvar). Previous research reported that polypeptides of 28kDa, 34kDa, and 40.5kDa are associated with thylakoid membrane protein (Tian et al., 2013; Huseynova et al., 2007). This can indicate that SA maintained an environment suitable for the function of critical integral proteins during drought stress via modulation of the ROS signal and adjusting chloroplasts and thylakoid membrane fluidity (Aldesuquy et al., 2018). These results provide a correlation between the synthesis of some molecular proteins and drought tolerance in SA-treated plants. The induction of dehydrin gene expression and protein accumulation for protecting cells from further dehydration and oxidative damage also has been reported (Sun et al., 2009). This is supported by several previous reports, in which SA treatment enhanced the levels of soluble protein, and the abundance of many enzymes related to the accumulation of polypeptides in wheat under stressful conditions. Treating plants with SA induced an increase in abundance of protein spots (including ribulose-1,5- bisphosphate carboxylase activase, two Rubisco large subunit-binding proteins, carbonic anhydrase) (Kang et al., 2012), and appearance of two de novo polypeptides (630 and 141 KDa) (Azooz et al., 2011) in order to cope with drought stress.

## 5 Conclusion

The results of this study support the hypothesis that Salicylic acid treatment might play an important role in modulating the physiological processes which eventually lead to protect plants under drought stress conditions. SA is of great potential to improve photosynthesis rate and chlorophyll content in wheat. SA maintained the integrity of the cell membrane and enhanced ROS scavenger activity, such as CAT, PPO, and SOD. It also increased the expression or intensity of certain polypeptides. Based on physiological differences between ecotypes in response to drought, "Baharband and Telvar" are considered as drought tolerant ecotypes (the highest photosynthetic performance), whereas "Fetrezamin and Gavdareh" are considered as drought-sensitive ecotypes (the highest cell membrane electrolytic leakage and MDA concentration and the lowest photosynthesis rate). In general, SA seems to be a promising method that could be used to ameliorate the negative effects of drought stress on wheat in areas where water deficit is a major constraint.

## Declarations

### Author contribution statement

Masoumeh Khalvandi: Performed the experiments; Analyzed and interpreted the data; Wrote the paper.

Adel Siosemardeh, Ebrahim Roohi: Conceived and designed the experiments; Analyzed and interpreted the data; Contributed reagents, materials, analysis tools or data.

Sara Keramati: Analyzed and interpreted the data; Wrote the paper.

### Funding statement

This research did not receive any specific grant from funding agencies in the public, commercial, or not-for-profit sectors.

### Data availability statement

Data will be made available on request.

### Declaration of interests statement

The authors declare no conflict of interest.

### Additional information

No additional information is available for this paper.
