# Supplementary material for: Salicylic acid alleviated the effect of drought stress on photosynthetic characteristics and leaf protein pattern in winter wheat
Source: Heliyon. 2021 Jan 7;7(1):e05908. doi: 10.1016/j.heliyon.2021.e05908 (PMC7809382; doi:10.1016/j.heliyon.2021.e05908)
Supplement: Supplemental Information [file mmc1.docx]

Supplemental Information

Salicylic acid alleviated the effect of drought stress on photosynthetic characteristics and leaf protein pattern in winter wheat

Masoumeh Khalvandi^*^, Adel Siosemardeh, Ebrahim Roohi, Sara Keramati

Contents:

The original SDS-PAGE gels


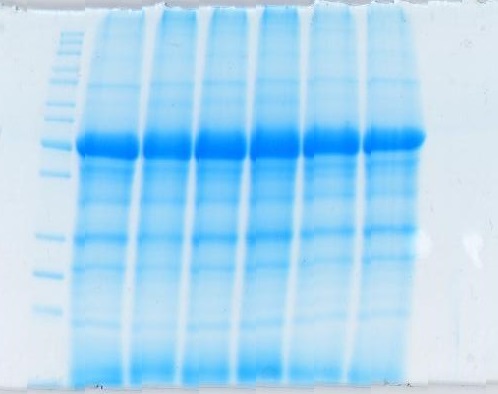


Supplemental Figure 1. Polypeptide patterns of soluble leaf protein extracts of six wheat ecotypes were grown under controlled conditions.


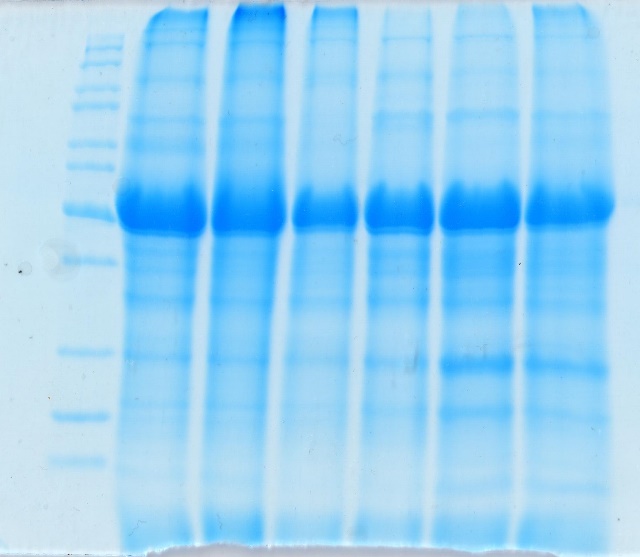


Supplemental Figure 2. Polypeptide patterns of soluble leaf protein extracts of six wheat ecotypes were grown under drought stressed conditions.


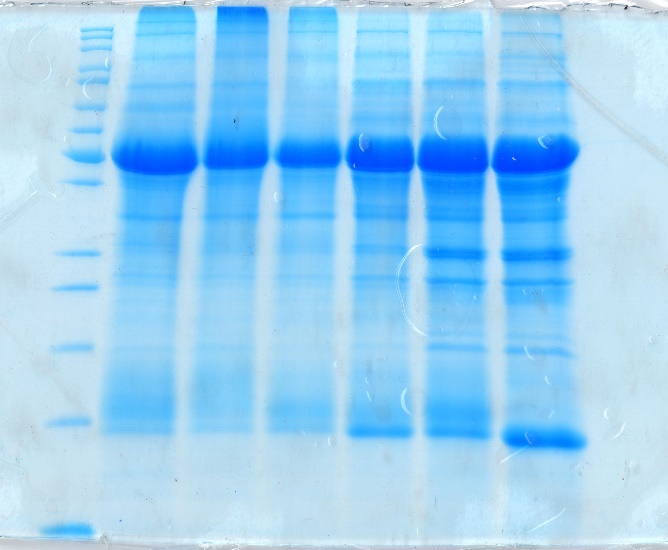


Supplemental Figure 3. Polypeptide patterns of soluble leaf protein extracts of drought-stressed wheat ecotypes that were sprayed with salicylic acid
